# Real-Time Edge-Prior Guided SegFormer for Robust Contour Extraction of Aggregate Particles in Conveyor-Belt Depth Maps

**DOI:** 10.3390/s26103196

**Published:** 2026-05-18

**Authors:** Jian Shen, Hanye Liu, Zhilin Chen, Xiangnan Zhao, Huijuan Yang

**Affiliations:** 1 School of Information Engineering, Yulin University, Yulin 719000, China; 18066105344@163.com (J.S.); chenzhilin147369@126.com (Z.C.); zxndewaterboard@163.com (X.Z.); 2 Computer Application, Yulin Vocational and Technical College, Yulin 719000, China; 15929409090@163.com

**Keywords:** depth map, contour extraction, boundary detection, SegFormer, Mix Vision Transformer, geometric prior, tolerance matching

## Abstract

Accurate contour extraction of aggregate particles from conveyor-belt depth maps is essential for downstream particle counting and size measurement, yet industrial depth data often contains weak discontinuities, missing values, and speckle-like noise. We propose a task-specific geometry-aware contour extraction framework that combines a compact SegFormer encoder with depth-derived priors, a lightweight local branch, edge-prior gated fusion, and full-resolution residual refinement. The input representation consists of normalized depth, Sobel gradient magnitude, and the absolute Laplacian response. On AGG_FULLDATA, the method achieves Optimal Dataset Scale (ODS), Optimal Image Scale (OIS), and Average Precision (AP) values of 0.9607/0.9716/0.9683 under the primary tolerance-based protocol (tol=1), while retaining an ODS of 0.6476 under strict pixel-exact matching. On External130, a test-only split collected under altered operating conditions using the same sensor, it reaches 0.9580/0.9734/0.9683 without retraining and consistently outperforms the MiT-only baseline. A rigid-object repeatability study based on 30 raw PLY scans shows a mean boundary deviation of 0.335 px, a within-1 px correspondence rate of 97.1%, and a coefficient of variation (CV) of equivalent diameter below 1%, supporting the practical meaning of tol=1. The full pipeline runs at 48.9 frames per second (FPS) with 3.71 M parameters on an NVIDIA GeForce RTX 4060 GPU. Broader robustness to separately controlled operating factors, environmental disturbances, and cross-device settings still requires validation.

## 1. Introduction

Precise delineation of particle contours in conveyor-belt depth maps serves as a fundamental input for measurement pipelines, including particle counting, size distribution estimation, and morphology analysis. In the specific context of this study, depth maps are acquired via a 2D/3D contour camera mounted above a conveyor belt. Unlike RGB imagery, this depth modality is less sensitive to illumination variations but provides minimal textural information; boundaries are indicated primarily by geometric discontinuities and may be degraded by sensor artifacts, missing depth measurements, and subtle depth transitions in densely packed scenes. Consequently, contour extraction poses both accuracy and engineering challenges. It involves missed or fragmented boundaries, while also requiring stability across operating conditions and compliance with runtime constraints. Classical edge detectors, such as the Canny operator, are computationally efficient and straightforward to deploy; however, their reliance on hand-crafted gradients and fixed thresholding renders them less robust in the presence of depth noise and pile-up. Deep learning-based edge detectors (e.g., HED, RCF, BDCN, DexiNed) improve accuracy by learning edge cues from data, yet many are optimized for RGB textures and often necessitate computationally intensive backbones. For measurements based solely on depth, a practical contour extractor must explicitly exploit geometric priors, preserve local discontinuities, and maintain computational efficiency. We propose a robust contour extraction module tailored to conveyor-belt depth maps. We use a compact SegFormer encoder (MiT-B0) as the global context branch and inject fixed geometric priors (Sobel gradient magnitude and Laplacian response) computed from the raw depth map. In parallel, a lightweight local CNN branch preserves fine-grained discontinuities that are easily lost in coarse transformer features. An edge-prior gated fusion block uses the gradient prior to modulate fused features, and a full-resolution residual refinement block corrects coarse predictions using both priors and the raw depth signal. The resulting pipeline is lightweight, straightforward to deploy, and designed for industrial settings rather than for introducing entirely new backbone components.

The data in this study come from a real conveyor-belt acquisition setup rather than from indoor RGB–D benchmarks. This distinction matters because industrial laser triangulation data exhibit depth-specific artifacts and operating constraints that differ from those of common public datasets. At the same time, the main split alone is not sufficient to support broad sensor-agnostic generalization claims. For this reason, we complement the main AGG_FULLDATA split with an External130 cross-session test-only set captured with the same sensor under altered acquisition conditions. Because the measuring rate and belt speed differ simultaneously between the two sessions, this split is treated as a limited same-sensor cross-session check rather than a factor-wise study of individual operating variables. The contribution of this study, therefore, lies in the task-specific integration, evaluation, and deployment-oriented design of the pipeline rather than in proposing a completely new backbone or edge detector.

The main contributions of this work are summarized as follows:

- We present a practical geometry-aware contour extraction pipeline for conveyor-belt depth maps by integrating a compact MiT-B0 encoder with explicit depth, gradient, and Laplacian cues, a lightweight local branch, gated fusion, and full-resolution refinement.
- We provide a transparent evaluation protocol for thin one-pixel contour annotations, reporting both a primary tolerance-based protocol (tol=1) to account for small local annotation shifts and a strict supplementary reference (tol=0) for pixel-exact matching, and we further support this choice with a rigid-object repeatability study based on repeated raw PLY scans under the same imaging setup.
- We supplement the main AGG_FULLDATA evaluation with an External130 cross-session test-only split and a DexiNed input-sensitivity analysis. These results provide a limited same-sensor check under a coupled measuring-rate/belt-speed shift, show that the proposed design remains competitive under this specific session shift, and indicate that the observed gains cannot be attributed solely to the pseudo-RGB input representation.
- We show that the resulting system achieves a favorable accuracy–efficiency trade-off on an NVIDIA GeForce RTX 4060 GPU (NVIDIA Corporation, Santa Clara, CA, USA), making it suitable for real-time conveyor-belt measurement scenarios.

The remainder of this paper is organized as follows. Section 2 reviews related work. Section 3 describes the dataset and annotation procedure. Section 4 presents the evaluation protocol. Section 5 details the proposed method. Section 6 summarizes the experimental setup. Section 7 reports the main results and discussion. Section 8 presents ablation studies. Section 9 concludes the paper.

## 2. Related Work

### 2.1. Particle Measurement and Depth Sensing

Depth sensing has been extensively employed to estimate particle size distributions in conveyor-based scenarios [1,2]. Using laser triangulation and 2D/3D contour cameras, recent studies have focused on predicting size distributions or segmenting particles directly from distance measurements [3,4]. For instance, dense convolutional neural networks have been applied to 3D laser triangulation data for predicting particle size distributions in construction and demolition waste recycling; instance segmentation has been performed on overlapping 3D-laser-triangulation particle flows; and online particle size analysis on industrial conveyors has been reported using deep networks [3,4,5]. Recent application-oriented segmentation studies have also explored adhesion-aware ore separation, Mask R-CNN-based aggregate identification and gradation analysis, improved Mask R-CNN variants for ore segmentation, and lightweight conveyor-belt coal/ore segmentation networks [6,7,8,9]. Multimodal fusion and semi-supervised segmentation have further been explored for material analysis when RGB cues are unreliable [10,11]. Taken together, these studies confirm the current interest in deployment-oriented particle analysis under industrial sensing constraints, while also showing that many recent methods target RGB/instance-mask segmentation or PSD estimation rather than one-pixel contour extraction from depth-only boundary maps. Precise boundary extraction remains a key component for morphology estimation and dimensional measurement. Beyond in-motion imaging, offline metrology systems, such as structured-light scanners and laser triangulation rigs, can compute the volume, surface area, and shape of aggregates [12,13]. However, these systems typically require stationary objects and controlled conditions; thus, boundary extraction from depth maps in dynamic settings remains necessary. Engineering studies in the field of construction materials often involve domain-specific acquisition setups, as the sensing geometry, calibration, and material handling conditions are integral parts of the overall measurement pipeline. For example, Wang et al. proposed a convolutional encoder–decoder model for the automatic segmentation of concrete aggregates in sedimentation images and reported strong segmentation accuracy on their test set [14]. Such application-driven research suggests that boundary quality and deployment constraints (including noise patterns, throughput, and camera placement) should be considered jointly. Consistent with this perspective, we validate our contour extractor under a fixed conveyor-belt depth-sensing setup and report both boundary metrics and efficiency.

### 2.2. Edge Detectors and Contour Detection

Classical edge operators, such as the Canny detector, combine gradient computation with non-maximum suppression and hysteresis thresholding [15]. Structured Forests learn decision-tree ensembles over hand-crafted features, providing a strong and efficient baseline for edge detection [16]. Deep edge detectors include HED with side-output supervision [17], RCF with richer multi-level features [18], BDCN with bidirectional cascade fusion [19], PiDiNet with an efficiency-focused design [20], and DexiNed with dense inception-style blocks [21]. RINDNet further targets edges induced by discontinuities in reflectance, illumination, surface normal, and depth [22]. Recent lightweight detectors, such as TEED, LED-Net, and LGLNet, also emphasize the accuracy–efficiency trade-off through very small parameter budgets or lightweight low-level feature extractors [23,24,25]. More recent accuracy-oriented architectures, such as Mask2Edge, continue to improve RGB edge benchmarks through masked attention and dynamic difference modeling [26]. This recent line of work is highly relevant to deployment-oriented systems, where contour quality must be balanced against model size and runtime. Because these methods are mainly developed for natural RGB imagery, only the subset that can be retrained and evaluated under a unified protocol on our private industrial depth data is included in the controlled benchmark; the resulting comparison protocol is described in Section 6. RGB-D edge cues have also been investigated for edge-based registration, highlighting the value of reliable depth discontinuities [27]. These methods are primarily developed for natural RGB images and may miss or merge boundaries when applied to depth-only inputs.

#### 2.2.1. Segmentation Backbones and Transformer Encoders

Encoder–decoder architectures, such as U-Net, remain the standard for dense prediction tasks [28,29]. Transformer backbones were popularized by the Vision Transformer (ViT), which applies token-based self-attention to image recognition [30]. SegFormer proposes a hierarchical Mix Vision Transformer (MiT) encoder coupled with a simple MLP decoder [31]. In this work, we adopt MiT-B0 as a compact context encoder and focus on geometry-aware boundary localization through explicit gradient priors and full-resolution refinement.

#### 2.2.2. Contour Evaluation Protocols

Contour detection is typically evaluated via precision–recall curves [32,33]. The Optimal Dataset Scale (ODS) represents the maximum F1 score on the test set across all thresholds; the Optimal Image Scale (OIS) is the average of the best F1 score for each image; and Average Precision (AP) is the area under the precision–recall curve. We report results using both tolerance-based matching (tol=1) and pixel-exact matching (tol=0) to characterize robustness against minor annotation ambiguity and strict localization accuracy, respectively.

#### 2.2.3. Depth Cues and Geometric Priors

Geometric encodings, such as HHA, improve boundary localization in RGB–D tasks [34]. Depth discontinuities and surface-normal variations guide networks to attend to geometric transitions. We normalize the depth map and compute gradient magnitudes and Laplacian responses via fixed Sobel and Laplacian kernels; these cues serve as input channels and gating priors in our fusion module. Prior work on RGB–D scene parsing emphasizes the importance of depth discontinuities and surface normals for segmentation [35,36], which motivates our use of gradient and Laplacian priors for contour extraction.

## 3. Dataset and Annotation

### 3.1. Acquisition System and Context

**Figure 1:**
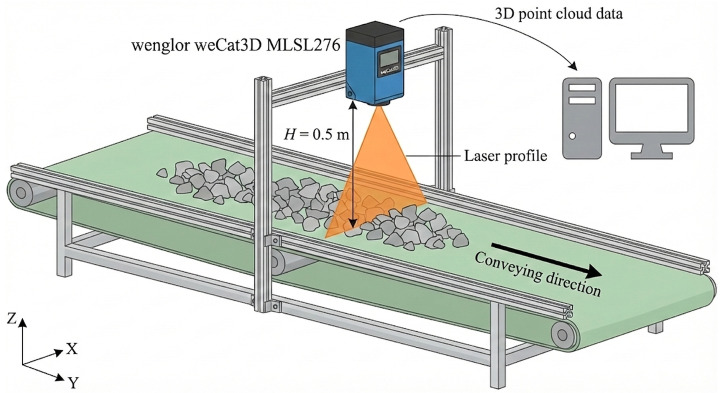
Depth capture system: a Wenglor MLSL276 2D/3D contour camera mounted approximately 0.5 m above the belt.

We collected the data using a conveyor-belt depth scanning setup designed for aggregate particles. A Wenglor MLSL276 2D/3D contour camera (wenglor sensoric elektronische Geräte GmbH, Tettnang, Germany) was mounted approximately 50 cm above the belt, projecting a laser line and measuring depth along the belt travel direction. Each frame yields a distance image with dimensions of 832×576 (height × width). The capture setup is illustrated in Figure 1.

**Figure 2:**
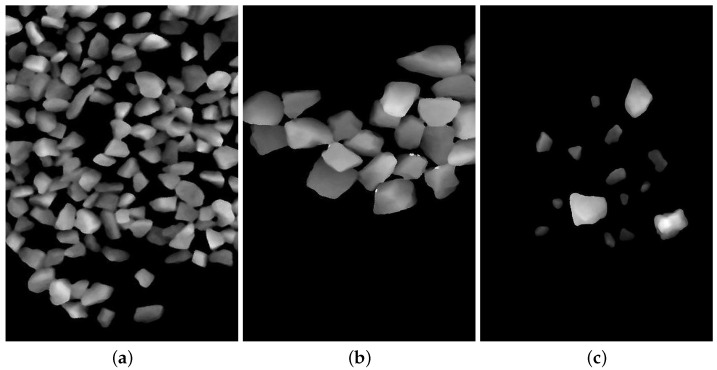
Representative depth maps from AGG_FULLDATA illustrating variations in pile-up density under the main acquisition setup. (**a**) High density. (**b**) Medium density. (**c**) Low density.

The main dataset, denoted as AGG_FULLDATA, was captured in Session A under a fixed camera pose and a nominal belt speed of approximately 0.21 m/s. The material consists of aggregate particles with a heterogeneous size distribution. The primary variation across frames is pile-up density, spanning high-, medium-, and low-density scenes. This variation affects the degree of occlusion, inter-particle contact, and local edge clutter (Figure 2). To examine robustness beyond the main split, we additionally collected an External130 cross-session test-only set in Session B using the same camera model but under altered acquisition conditions. In particular, Session B uses a measuring rate of 250 Hz and a belt speed of approximately 0.26 m/s, whereas the main split uses 200 Hz and approximately 0.21 m/s. Because these two operating parameters are changed simultaneously, External130 does not isolate whether performance changes are driven by along-track spatial sampling/resolution effects associated with belt speed or by measuring-rate-related sensor-noise characteristics. This study therefore provides a same-sensor cross-session evaluation under changed operating conditions rather than cross-device validation. Table 1 summarizes the acquisition conditions for the two splits.

#### Depth Map Generation

The raw point cloud is projected into a 2D depth map representing the distance from the sensor to each surface point. These maps serve as the inputs to our model.

### 3.2. Annotation Protocol

**Figure 3:**
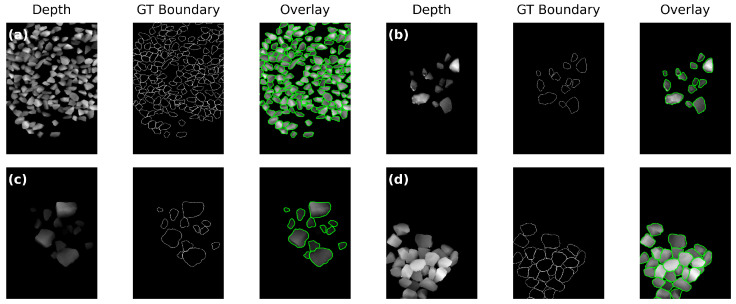
Representative examples of one-pixel contour annotations in AGG_FULLDATA. (**a**) Dense aggregate sample. (**b**) Sparse aggregate sample. (**c**) Low-density partial-frame sample. (**d**) High-density partial-frame sample. For each sample, the panels show the depth map, the ground-truth contour, and the overlay from left to right.

For all images, we manually annotated thin binary contour maps. We traced only the outer boundary of each particle, without applying smoothing or dilation. Due to display scaling, limited cursor precision, and subtle depth variations, manual tracing inevitably introduces slight shifts (typically one pixel). Therefore, we employ tolerance-based matching with tol=1 as the primary evaluation protocol to account for minor annotation ambiguity, and we additionally report tol=0 results as a strict reference for pixel-exact localization. Figure 3 shows representative depth maps together with one-pixel contour annotations and their overlays.

#### 3.2.1. Dataset Splits

For AGG_FULLDATA, we use a fixed split of 1200 training images, 200 validation images, and 200 test images. The additional External130 set contains 130 images with corresponding contour annotations and is used strictly as a test-only set. No model selection or hyperparameter tuning is performed on External130.

#### 3.2.2. Data Access Note

The depth maps were collected in a controlled industrial setup and are subject to project-specific data-sharing restrictions, so the raw data are not publicly released. To support verifiable evaluation and protocol reuse, upon acceptance we will release a reproducibility package containing: (i) the evaluation script that implements the threshold sweep (0.01–1.01, step 0.01) and tolerance-based matching (tol∈{0,1}); (ii) fixed file lists that define the exact AGG_FULLDATA and External130 test images used for all reported numbers; (iii) the released test-set contour masks for these two evaluation splits; (iv) the per-method test-set probability maps exactly as evaluated to produce the tables and figures in this paper; and (v) the trained checkpoints for HybridMiT and the compared learning-based baselines. Access to the remaining full dataset, including unreleased depth maps and annotations outside the released test splits, may be granted to qualified researchers under a non-disclosure agreement. Models are trained on the AGG_FULLDATA training split, selected on the validation split, and evaluated on the AGG_FULLDATA test split and the External130 test-only split.

## 4. Evaluation Protocol

### 4.1. Probability Output and Binarization

Learning-based methods produce a probability map $P\in R^{H\times W}$; Canny edges are treated as binary probabilities. Given a threshold τ, a binary prediction is obtained by

(1) $$B_{\tau }\left(x\right)=1\left(P(x)\geq \tau \right),\;x\in \left\{1,\ldots ,H\right\}\times \left\{1,\ldots ,W\right\},$$

where 1(·) denotes the indicator function. We sweep the threshold over a discrete set

(2) $$\begin{array}{cc} T & =\{\tau_{\mathrm{start}}+k\Delta \tau |k=0,1,\ldots ,100\},\\ \tau_{\mathrm{start}} & =0.01,\;\Delta \tau =0.01,\;\tau_{\mathrm{end}}=1.01, \end{array}$$

i.e., thresholds are swept from 0.01 to 1.01 with a step size of 0.01. The final threshold point (τ=1.01) yields an empty prediction map by construction and serves as a zero-recall endpoint for the precision–recall curve; when $\sum B_{\tau }=0$, precision is defined by convention and does not affect the reported ODS/OIS maxima.

### 4.2. Tolerance Matching and Metrics

We evaluate predictions using a tolerance-based boundary matching scheme to account for minor annotation ambiguity in thin contours. Let *y* denote the binary ground-truth contour and $B_{\tau }$ denote the thresholded prediction at τ. We construct dilated masks $y^{\left(tol\right)}=\mathrm{Dilate}\left(y,tol\right)$ and $B_{\tau }^{\left(tol\right)}=\mathrm{Dilate}\left(B_{\tau },tol\right)$, where tol is measured in pixels. Matched true positives for precision are computed as $TP_{p}=\sum \left(B_{\tau }⊙y^{\left(tol\right)}\right)$, while matched true positives for recall are computed as $TP_{r}=\sum \left(y⊙B_{\tau }^{\left(tol\right)}\right)$. Precision and recall are then defined as

(3) $$\mathrm{Precision}=\frac{TP_{p}}{\sum B_{\tau }},\;\mathrm{Recall}=\frac{TP_{r}}{\sum y},$$

In degenerate cases where $\sum B_{\tau }=0$, we define Precision as 1 and Recall as 0 (thus BF1=0), consistent with common precision–recall implementations. The boundary F1 score (BF1) is calculated as

(4) $$\mathrm{BF}1=\frac{2\;\mathrm{Precision}\times \mathrm{Recall}}{\mathrm{Precision}+\mathrm{Recall}}.$$

We report ODS (Optimal Dataset Scale), OIS (Optimal Image Scale), and AP (Average Precision) derived from the precision–recall curve. Unless otherwise stated, tol=1 pixel; tol=0 corresponds to pixel-exact matching.

#### 4.2.1. Auxiliary Metric

As an auxiliary metric, we also report BF1 at 0 pixels (BF1@0). All baselines and ablations use the same threshold sweep and tolerance-matching protocol (default tol=1).

#### 4.2.2. Why tol=1 Is the Primary Protocol

The primary protocol in this paper uses a 1-pixel tolerance (tol=1) because the contour annotations are one pixel wide and small local shifts are unavoidable in manual tracing. In practice, the exact centerline selected by an annotator may differ by one pixel even when the same particle boundary is interpreted consistently. This issue is particularly pronounced in thin contours, weak depth transitions, and crowded regions where several particles touch. Moreover, in laser-triangulation depth maps, the apparent transition near a particle boundary may be broadened by several factors, including the finite laser-line width, surface roughness, reflectance variations, range quantization, and rasterization of the scanned profile into the image grid. We therefore do not interpret a one-pixel disagreement as necessarily indicating a meaningful boundary error. For this reason, we use tol=1 as the main evaluation protocol and regard it as a practical treatment of annotation ambiguity and image-formation uncertainty rather than as a claim of universal physical necessity. We additionally report pixel-exact results (tol=0) as a stricter supplementary reference. This second protocol is useful because it reveals how sensitive a method is to one-pixel shifts and provides a complementary perspective alongside the tolerance-based scores. Tolerance-based evaluation is standard in contour detection benchmarks [32,33]. In our setting, the combination of tol=1 and tol=0 provides a balanced view of contour continuity and strict localization. To further ground the practical meaning of this protocol in the present setup, we additionally conducted a rigid-object repeatability study using three simple rigid targets (triangle, cylinder, and cube), each scanned ten times under the same Session A camera configuration. Starting from the raw PLY outputs, we rasterized the scans in the native scan plane, removed the conveyor background, extracted the outer target boundary, and rigidly aligned repeated scans in image space. As summarized later in Section 7.3, the overall pooled mean boundary deviation is 0.335 px and the pooled P95 distance is 1.000 px. In addition, 97.1% of boundary correspondences fall within 1 px, while the CV of equivalent diameter remains below 1% overall. These observations do not establish a universal physical law, but they do support tol=1 as a practical primary protocol for the present one-pixel annotation scheme and laser-triangulation imaging configuration.

## 5. Proposed Method

### 5.1. Overview

**Figure 4:**
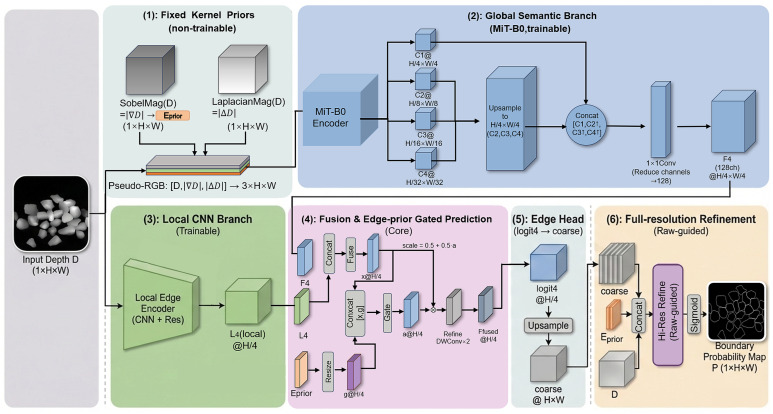
Overall architecture of HybridMiT: fixed-kernel geometry priors form a pseudo-RGB input for a MiT-B0 encoder; a lightweight local branch complements high-frequency detail; a prior-guided gated fusion and a full-resolution residual refinement produce the final contour probability map. Arrows indicate the forward flow of images, features, edge priors, and probability maps between modules.

As illustrated in Figure 4, our network integrates global context, local geometry, and explicit edge priors. The input depth map is normalized and concatenated with gradient magnitude and Laplacian channels to form a three-channel tensor. A global branch based on a lightweight MiT-B0 encoder extracts multi-scale contextual features, while a local branch captures high-frequency detail. These features are fused through a gating mechanism controlled by a Sobel-derived prior. Two prediction stages, including a full-resolution residual refinement, generate the final probability map. For clarity, the Sobel magnitude prior is denoted as $E_{\mathrm{prior}}$ (referred to as *g* in the text) in Figure 4. The depth input to the refinement stage corresponds to the normalized depth $\hat{D}$. We adopt the MiT-B0 encoder from SegFormer as the context backbone and redesign the head with local fusion and full-resolution refinement for one-pixel contour extraction.

#### 5.1.1. Input Construction and Gradient Prior

Given a raw depth map *D*, we first normalize it as

(5) $$\hat{D}=\frac{D-\min(D)}{\max(D)-\min(D)+\varepsilon },\;\varepsilon =10^{-6}.$$

We then compute a Sobel gradient magnitude and an absolute Laplacian response using fixed (non-trainable) kernels:

(6) $$\begin{array}{cc} S & =\sqrt{{\left(K_{x}*\hat{D}\right)}^{2}+{\left(K_{y}*\hat{D}\right)}^{2}+\varepsilon },\\ L_{\mathrm{abs}} & =\left|K_{\mathrm{lap}}*\hat{D}\right|, \end{array}$$

where “∗” denotes the convolution operation. These maps are normalized to [0,1] (per-image min–max) and stacked to form a three-channel tensor $X\in R^{3\times H\times W}$. Given that MiT-B0 is pre-trained on ImageNet RGB images, directly feeding depth-only values introduces an input-domain mismatch. Following the standard SegFormer preprocessing, we apply ImageNet mean/std normalization to *X* before feeding it into the MiT encoder. Since *X* is a pseudo-RGB stack of geometric cues rather than natural colors, this normalization is used only to keep the input scale compatible with the pre-trained weights rather than to imply any statistically matched RGB distribution. ImageNet-pretrained MiT-B0 weights, therefore, serve only as an initialization choice for this pseudo-RGB input. However, this study does not include a controlled comparison between random initialization and ImageNet pretraining. Therefore, we do not attempt to isolate or quantify the independent contribution of pretraining to the final performance. The reported improvements should be interpreted as the combined effect of the proposed edge-prior-guided architecture, the geometric input representation, the fusion and refinement design, and the adopted initialization strategy. A systematic investigation of random initialization, ImageNet pretraining, and depth-domain pretraining is left for future work. Another limitation concerns the use of ImageNet-pretrained MiT-B0 weights for a pseudo-RGB input composed of normalized depth, Sobel gradient magnitude, and Laplacian response. Although ImageNet pretraining provides a practical initialization for the encoder, the learned filters originate from natural photometric textures and color statistics, whereas the proposed input channels represent deterministic geometric measurements and their derivatives. This domain gap may limit the ability of the pretrained feature extractor to fully exploit depth-specific structures, especially for fine boundary cues affected by scanning noise or local surface discontinuities. Future work will investigate depth-domain pretraining, self-supervised pretraining on unlabeled conveyor-belt depth maps, and reconstruction or contrastive objectives tailored to geometric modalities. The normalized Sobel magnitude is also used as the edge prior *g* for feature gating and full-resolution refinement.

#### 5.1.2. Global Feature Extraction Based on MiT-B0

The global context branch in Figure 4 employs the Mix Vision Transformer (MiT-B0) encoder from SegFormer [31]. The MiT architecture uses overlapping patch embeddings and a four-stage hierarchy. Given the normalized three-channel input, MiT outputs feature maps $\left\{C_{1},C_{2},C_{3},C_{4}\right\}$ at resolutions {H/4,H/8,H/16,H/32} with channel dimensions {32,64,160,256}. To obtain a single quarter-resolution context representation, we bilinearly upsample $C_{2},C_{3},C_{4}$ to the $C_{1}$ resolution (H/4×W/4), concatenate them with $C_{1}$, and compress the resulting 512-channel tensor using a 1×1 convolution followed by batch normalization and GELU activation. The output is the global feature $F_{g}\in R^{128\times H/4\times W/4}$.

#### 5.1.3. Local Branch and Prior-Guided Fusion

To preserve high-frequency geometric detail that may be attenuated by downsampling, we incorporate a shallow local branch that operates directly on the normalized depth input. The local encoder applies two stride-2 convolutions (1→32 at H/2, then 32→64 at H/4), followed by a residual block at 64 channels. A 1×1 projection produces $F_{l}\in R^{64\times H/4\times W/4}$. All activations in this branch use GELU. We fuse the global and local features via concatenation and a lightweight fusion block. Specifically, $\left[F_{g},F_{l}\right]$ is projected to 128 channels with a 1×1 convolution, followed by batch normalization, GELU, and a depthwise-separable convolution (DWConv). To inject explicit boundary evidence, we compute a channel-wise gate from the concatenation of the fused feature and the resized edge prior *g*. The gate is produced by two 1×1 convolutions with a GELU nonlinearity and a final sigmoid. Rather than applying hard masking, we use a conservative modulation F←F⊙(0.5+0.5gate), followed by two DWConv layers for refinement. The output is the fused quarter-resolution feature $F_{f}\in R^{128\times H/4\times W/4}$.

#### 5.1.4. Two-Stage Prediction and Full-Resolution Refinement

We predict coarse logits $z_{4}$ at quarter resolution from $F_{f}$ using a 3×3 convolution, batch normalization, GELU, and a final 1×1 convolution. The coarse logits are upsampled to full resolution via bilinear interpolation to obtain $z_{↑}$. To improve pixel-level alignment, we apply a full-resolution residual refinement module. The refinement input concatenates the upsampled logits, the full-resolution edge prior, and the normalized depth, forming a tensor in $R^{3\times H\times W}$. A small conv–BN–GELU stack predicts a residual δ, which is added to the upsampled logits:

(7) $$z_{\mathrm{final}}=z_{↑}+\delta ,\;P=\sigma \left(z_{\mathrm{final}}\right),$$

where σ denotes the sigmoid function. We do not apply any morphological post-processing.

#### 5.1.5. Loss Function and Auxiliary Supervision

Contours occupy only a small fraction of each image, leading to severe class imbalance. We therefore combine a weighted binary cross-entropy (BCE) loss with a Dice loss. Let $y\in {\left\{0,1\right\}}^{H\times W}$ be the one-pixel ground-truth contour map. We dilate and erode *y* with a kernel of size k=2r+1 and radius r=2, yielding $y^{\mathrm{dil}}$ and $y^{\mathrm{ero}}$. The band mask is defined as

(8) $$B=\mathrm{clip}\left(y^{\mathrm{dil}}-y^{\mathrm{ero}},0,1\right).$$

To distinguish near-boundary background from the positive contour pixels, let $B_{0}=B⊙\left(1-y\right)$ denote the near-boundary background band. We aim to focus the model on positive contour pixels while avoiding over-penalization of the immediate background, which usually contains annotation jitter. We define the weights as:

(9) $$\begin{array}{cc} w_{+} & =1+\alpha B,\\ w_{-} & =\gamma +\beta B_{0},\\ W & =w_{+}\;y+w_{-}\;\left(1-y\right), \end{array}$$

with α=2.0, γ=0.7, and β=0. We explicitly set β=0 based on empirical observations: the labels are one pixel wide, while the observed depth transition around a true boundary may extend over neighboring pixels because of annotation jitter, weak discontinuities, and the sensor-to-image rasterization process. Penalizing the immediate near-boundary background (i.e., setting β>0) made the predictions overly thin and fragmented in our experiments, which degraded contour continuity. Therefore, we disable the penalty for the near-boundary background band to improve robustness against minor local shifts. Given logits *z* and labels *y*, the weighted BCE loss is

(10) $$L_{\mathrm{BCE}}=-\frac{1}{HW}\sum_{x}W\left(x\right)\left[y\left(x\right)\log\sigma \left(z\left(x\right)\right)+\left(1-y(x)\right)\log\left(1-\sigma (z(x))\right)\right].$$

Let p=σ(z) be the sigmoid activation; the Dice loss is

(11) $$L_{\mathrm{Dice}}=1-\frac{2\sum (p⊙y)}{\sum p+\sum y+\varepsilon },$$

where ε is a small constant to avoid division by zero. The main loss combines these terms:

(12) $$L_{\mathrm{main}}=L_{\mathrm{BCE}}+L_{\mathrm{Dice}}.$$

If auxiliary supervision is enabled, we compute an auxiliary loss on the coarse logits $z_{4}$ and form the total loss as

(13) $$L=L_{\mathrm{main}}+\lambda \;L_{\mathrm{aux}},\;\lambda =0.4.$$

Auxiliary supervision is enabled by default (λ=0.4) unless otherwise stated.

## 6. Experimental Setup

### 6.1. Data Splits and Evaluation Protocol

All models are trained on AGG_FULLDATA, an industrial depth dataset collected from an on-site conveyor-belt aggregate processing line for this study. AGG_FULLDATA contains 1200 training images, 200 validation images, and 200 test images. We additionally evaluate on the External130 cross-session test-only split described in Section 3. For fair comparison, all methods are selected by validation ODS on AGG_FULLDATA under the same threshold sweep (0.01–1.01 with a step of 0.01) and the same primary tolerance-based protocol (tol=1). Unless otherwise stated, we report both tol=1 and tol=0 on the AGG_FULLDATA test split, and we also report cross-session results on External130 without any retraining or hyperparameter tuning on that split.

### 6.2. Implementation Details and Training Strategy

Our implementation is based on PyTorch 2.0. We use the AdamW optimizer and enable automatic mixed precision (AMP). All depth maps are resized to ensure that both spatial dimensions are divisible by 32, matching the MiT downsampling stages. The context encoder is initialized with publicly released MiT-B0 pre-trained weights (SegFormer) and is then trained jointly with the remaining modules. We adopt a step-based training schedule with early stopping. Specifically, we evaluate on the validation set every 1000 steps using the tolerance-based protocol (tol=1); the checkpoint with the highest validation ODS is retained as the final model, and training terminates when no improvement is observed for a fixed patience window.

#### 6.2.1. Baseline Adaptation Protocol

All compared learning-based baselines are trained and evaluated as independent models; we do not insert HybridMiT components into them. For RGB-oriented edge detectors (HED, RCF, BDCN, DexiNed, PiDiNet, and TEED), the default comparison uses the common adaptation of replicating the normalized single-channel depth map to three channels so that the original network interface remains unchanged. For the MiT-only baseline, we retain the same pseudo-RGB input construction used by our method and remove the local branch, the gated fusion block, and the full-resolution refinement block; this isolates the value of the added modules more directly. For the classical edge-detector baselines that rely on VGG backbones (HED, RCF, and BDCN), we initialize the backbone with ImageNet-pretrained VGG16 weights, following the standard practice of the original implementations. For the U-Net baseline, we adopt a ResNet-50 encoder initialized with ImageNet-pretrained weights. Most learning-based baselines are trained under the same step-based budget; if a method does not provide an official pre-trained checkpoint compatible with our single-channel depth input, we train it under the same unified protocol and report the setting explicitly. To broaden the deployment-oriented comparison with a recent lightweight reference, we include TEED in the quantitative benchmark because its official implementation can be retrained and evaluated consistently under the same protocol. LED-Net, LGLNet, and Mask2Edge are therefore discussed in Section 2 only, as a controlled retraining pipeline under the same protocol was not available for this study. For the very lightweight TEED baseline, we use random 300×300 crops from the original 832×576 depth images, consistent with the default compact square-input training style of the official TEED code, while keeping the same train/val/test partition, model-selection rule, and test-time evaluation protocol.

#### 6.2.2. Classical Watershed Baseline

**Figure 5:**
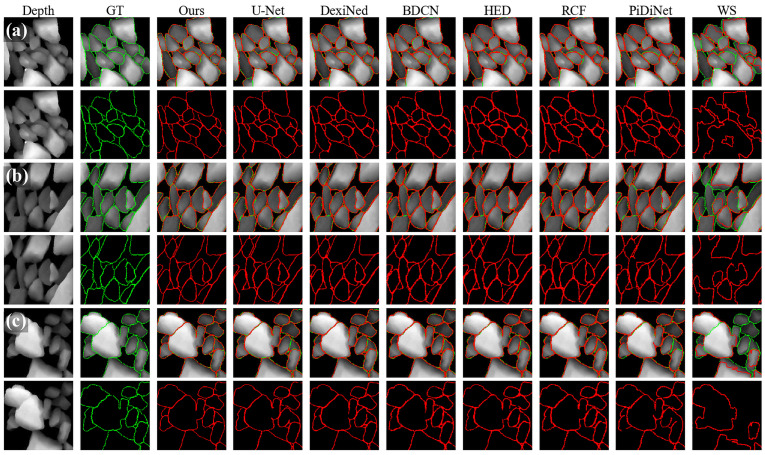
Qualitative boundary overlays on AGG_FULLDATA. (**a**) Representative crowded particle case. (**b**) Close-up of touching-particle boundaries. (**c**) Additional close-up with strong local boundary contacts. In each subfigure, columns from left to right show the depth input, ground truth (GT), and predictions from representative methods including our method (Ours), several learning-based baselines, and a classical watershed segmentation (WS). The watershed baseline uses the immersion-based watershed algorithm [37] and is shown for qualitative comparison only.

For qualitative comparison, we include a classical watershed segmentation baseline. We follow the immersion-based watershed algorithm described by Vincent and Soille [37]: the normalized depth map is smoothed, its gradient magnitude is computed, and a watershed transform is applied. The watershed lines are converted to a binary boundary map and then rescaled to form a probability map. Since this baseline is not optimized for one-pixel contour detection and tends to over-segment touching particles, we report it only in the qualitative comparisons (Figure 5) and omit it from the quantitative tables.

In our implementation and efficiency benchmark, the fixed-kernel priors can be computed either as part of the data pipeline or on-the-fly within the model; when measured, the reported inference time includes all operations executed in the forward pass. We employ only simple geometric augmentation: random horizontal and vertical flips applied jointly to the input depth maps and their contour labels, each with a probability of 0.5. As the raw industrial data remain private, we do not release the original depth maps publicly. Upon acceptance, we will release the evaluation code, the fixed file lists, the released test-set contour masks, the test-set probability maps, and the trained checkpoints described in the Data Availability Statement to facilitate reproducibility on both AGG_FULLDATA and External130.

## 7. Results and Discussion

### 7.1. Main Comparison on AGG_FULLDATA

Table 2 reports the main comparison on AGG_FULLDATA under the primary tolerance-based protocol (tol=1). Table 3 reports the strict pixel-exact reference (tol=0). We include both protocols because the annotations are one pixel wide and small local tracing shifts are unavoidable, but it is also informative to examine how sharply performance changes when no tolerance is allowed. Among the recent lightweight detectors, TEED is included here as a representative efficiency-oriented reference evaluated under the same unified retraining and test protocol as the other learnable baselines.

Under tol=1, the proposed model attains the highest ODS, OIS, and AP on AGG_FULLDATA. DexiNed represents the strongest baseline among the compared methods, remaining very close in ODS and AP, while the VGG-based detectors fall further behind. TEED, the most recent lightweight detector in this benchmark, does not reach the top ODS/OIS performance levels observed in this benchmark but provides an efficiency-oriented reference point under the same protocol. The MiT-only baseline benefits substantially from the addition of the local branch, gated fusion, and full-resolution refinement, indicating that the gain does not come from the MiT backbone alone.

**Figure 6:**
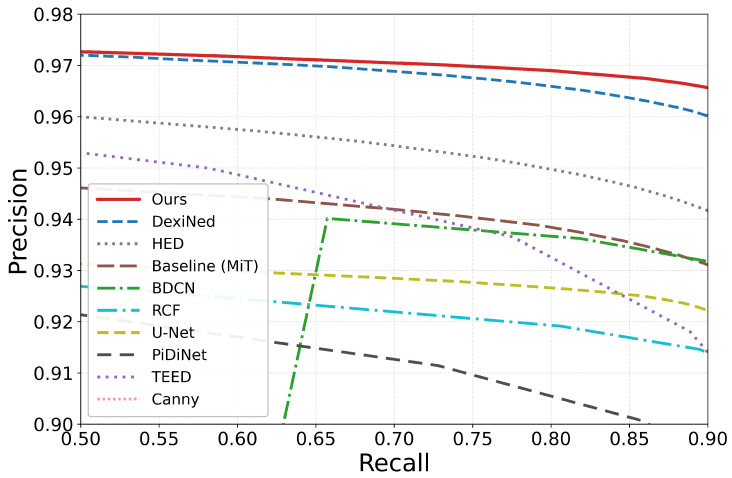
Locally zoomed precision–recall comparison on the AGG_FULLDATA test set under the same protocol as Table 2. The view focuses on the high-performance operating region where the strongest methods overlap, including TEED as the lightweight reference baseline.

Figure 6 shows that the improvement is not restricted to a single threshold. For readability, the plot presents a zoomed view of the high-performance region; it remains consistent with Table 2 and places the separation among the strongest methods easier to inspect.

### 7.2. Dexined Input-Sensitivity Analysis

Because the compared methods use different input encodings, it is informative to examine how sensitive a strong baseline is to the pseudo-RGB representation adopted by HybridMiT. To this end, we re-trained DexiNed with two input modes: (i) the default replicated-depth input (Rep3), and (ii) the same Depth+Sobel+|Laplacian| pseudo-RGB input used by HybridMiT (DSL3). The baseline architecture was otherwise left unchanged. Table 4 summarizes this input-sensitivity analysis.

These results show that the stronger pseudo-RGB input does not automatically improve DexiNed in this setting. Instead, DexiNed performs worse with DSL3 than with the simpler replicated-depth input. These results indicate that the advantage of HybridMiT is not solely attributable to the input representation. A more precise interpretation is that input representation and network architecture are coupled, and the proposed design benefits from this representation more effectively than DexiNed does.

### 7.3. Strict Pixel-Exact Reference

Under the strict pixel-exact protocol, performance drops for all methods. This behavior is expected for one-pixel annotations and is best considered alongside the tolerance-based scores. Our method attains the highest ODS and OIS, while DexiNed attains the highest AP.

#### Rigid-Object Repeatability Study

**Figure 7:**
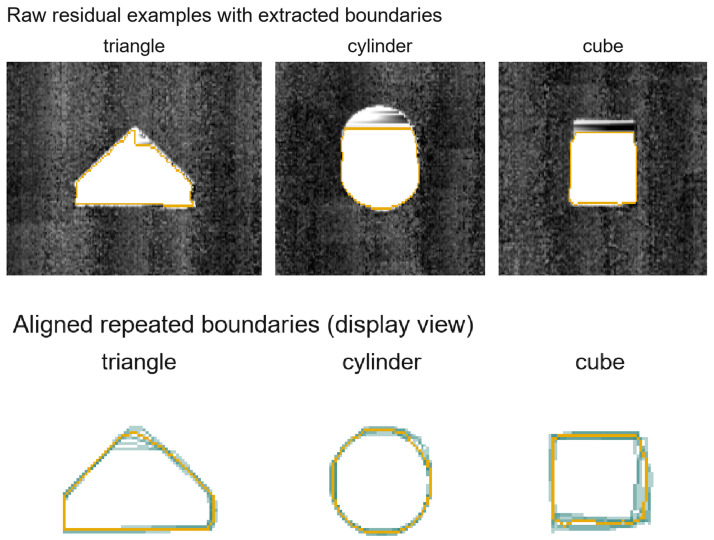
Rigid-object repeatability study based on 30 raw PLY scans acquired under the same Session A setup. The upper row shows representative residual crops after conveyor-background removal with the extracted outer boundaries overlaid. The lower row shows the corresponding aligned repeated boundaries for the triangle, cylinder, and cube targets. The lower row uses shape-regularized projected silhouettes for visual clarity only; the quantitative statistics are computed from the native scan-plane extraction.

To quantify the practical meaning of the tol=1 versus tol=0 gap under the same imaging setup, we conducted an additional repeatability study using three convex rigid targets (triangle, cylinder, and cube), each scanned ten times under the same Session A configuration. Starting from the raw PLY outputs, we rasterized each scan in the native scan plane, estimated and removed the conveyor background, extracted the outer target region from the depth residual, and aligned the repeated scans using 2-D rigid registration. Figure 7 shows representative raw residual crops together with the aligned repeated boundaries. For visual clarity, the lower row uses shape-regularized projected silhouettes for display only. The quantitative values in Table 5 are computed from the native scan-plane extraction rather than from the display-regularized silhouettes.

The measured repeatability variation is low but nonzero. Over all 30 scans, exact pixel overlap remains substantially lower than within-1 px agreement (70.1% vs. 97.1%), confirming that tol=0 is much more sensitive to local one-pixel shifts. At the same time, the CV of equivalent diameter remains below 1% overall, suggesting that these local boundary fluctuations have limited impact on coarse downstream size descriptors in this setup. In summary, tol=0 remains a useful strict supplementary reference, whereas tol=1 better reflects contour continuity and engineering tolerance for the present annotation scheme and laser-triangulation depth-imaging configuration.

### 7.4. Cross-Session Evaluation on External130

To examine robustness beyond a single acquisition session, we evaluate the final checkpoints on External130 without retraining. External130 is a same-sensor cross-session test-only split collected under altered acquisition conditions (Section 3), so we treat it as a limited robustness check rather than as a second development benchmark. It should also be noted that External130 changes two acquisition parameters simultaneously relative to the main Session A split: the measuring rate is increased from 200 Hz to 250 Hz, and the belt speed is increased from approximately 0.21 m/s to 0.26 m/s. These two factors are, therefore, confounded in the current cross-session evaluation. As a result, the observed performance differences on External130 cannot be uniquely attributed to either the change in along-track spatial sampling caused by belt speed or the change in sensor response and noise characteristics associated with the measuring rate. The External130 experiment should, therefore, be interpreted as a practical cross-session robustness test under a coupled operating-parameter shift rather than as a controlled factor-isolation study. Table 6 summarizes the results.

Several observations follow. These results do not establish universal superiority over DexiNed under session shift. Instead, they show that HybridMiT remains close to the strongest baseline on this limited external split without retraining. DexiNed is slightly better in ODS under both settings and better in AP, with the AP gap becoming larger under the strict pixel-exact reference, whereas HybridMiT achieves higher OIS under both tolerance settings. The full model still outperforms the MiT-only baseline, which supports the value of the geometry-aware additions even when the operating conditions change. The weaker ODS/OIS of TEED on External130 is consistent with its smaller capacity and confirms its role as a lightweight reference rather than a competitive contour extractor in this setting. When considered together with Table 7, these results indicate a favorable deployment-oriented trade-off: HybridMiT is markedly smaller and faster than DexiNed, remains very close to it on this limited cross-session split, and performs best on the main AGG_FULLDATA benchmark. Overall, External130 provides a limited same-sensor robustness check under a coupled change of operating parameters, rather than evidence of universal cross-sensor or environmental robustness.

### 7.5. Efficiency and Qualitative Discussion

Unless otherwise stated, efficiency is measured under single-image inference (batch size 1) with the network in evaluation mode and gradient computation disabled, at a fixed input resolution of 832×576. We run 50 warm-up iterations followed by 200 timed iterations and report the mean latency per iteration. Table 7 shows that HybridMiT maintains a strong efficiency profile despite the added geometry-aware components. TEED is by far the smallest and fastest model in this benchmark, but its contour accuracy in Table 2 and Table 6 remains clearly below that of the strongest baselines under both the primary and strict references, and this gap persists on the external split. For deployment, this indicates that TEED occupies a different accuracy–efficiency operating point rather than serving as a direct replacement when local boundary fidelity matters for downstream counting and size estimation. Compared with DexiNed, the proposed model is markedly lighter and runs at roughly 2.4 × higher FPS on the same GPU. Combined with Table 2 and Table 6, this indicates a favorable deployment-oriented trade-off: HybridMiT performs best on the main benchmark, remains close to DexiNed under the limited External130 session shift, and incurs significantly lower computational and deployment costs. This combination makes the method well suited to conveyor-belt systems where both accuracy and throughput are critical.

Figure 5 visualizes typical predictions on AGG_FULLDATA. Qualitatively, the proposed model often yields more continuous contours around touching particles and fewer spurious responses in flat depth regions. These observations are intended to illustrate the numerical results rather than to serve as additional quantitative evidence of contour continuity or false-positive suppression.

## 8. Ablation Studies

We perform ablation on the key components of the proposed framework: (i) the geometric prior inputs (Sobel gradient magnitude and Laplacian response), (ii) the local convolutional branch, (iii) the edge-prior gated fusion block, and (iv) the full-resolution residual refinement module. All variants are trained with the same budget as Table 8 and evaluated on the same AGG_FULLDATA test split.

Removing geometric priors or the local branch consistently reduces ODS and AP under both tolerance settings. Removing the full-resolution refinement module causes the largest overall degradation, indicating that pixel-level contour refinement at the original image resolution is important in this task. The isolated gate ablation shows only a modest difference between the full model and the variant without gate. At tol=0, the variant without gate is only marginally higher in AP (0.5736 vs. 0.5735), whereas the full model remains best in ODS and OIS under both tolerance settings. We therefore interpret the gate as a cooperative design choice rather than as the dominant source of the performance gain. The main contribution of the full model comes from the combined geometry-aware design, especially the explicit priors, the local branch, and the full-resolution refinement stage. Table 9 summarizes these ablation results.

## 9. Conclusions

We present a practical and deployment-oriented geometry-aware contour extraction framework for conveyor-belt depth maps of aggregate particles. The method combines a lightweight MiT-B0 context encoder with explicit depth/gradient/Laplacian priors, a local convolutional branch, gated fusion, and full-resolution residual refinement. On the main AGG_FULLDATA split, it reaches ODS=0.9607, OIS=0.9716, and AP=0.9683 under the primary tolerance-based protocol (tol=1), while retaining ODS=0.6476 under the strict pixel-exact reference (tol=0). On the External130 cross-session test-only split, the method remains close to the strongest baseline without retraining, reaching 0.9580/0.9734/0.9683 for ODS/OIS/AP under tol=1, while consistently outperforming the MiT-only baseline. DexiNed remains slightly stronger in ODS/AP on this external split, whereas HybridMiT attains higher OIS and a much better efficiency profile. Taken together, these results demonstrate that HybridMiT performs best on the primary AGG_FULLDATA benchmark. It also remains competitive under the limited same-sensor cross-session shift evaluated in External130.

### Limitations and Future Work

This study has several limitations. First, the external validation is cross-session rather than cross-device; all data come from the same sensor family and the same general conveyor-belt scenario. Moreover, the measuring rate and belt speed are changed together in External130, so this split cannot separate the effects of along-track spatial sampling from measuring-rate-related sensor-noise changes. Public RGB edge-detection benchmarks such as BSDS500 differ from the present task in imaging modality (RGB vs. depth-only), annotation format (multi-label semantic edges vs. one-pixel particle contours), and evaluation target (generic edge quality vs. closed-contour completeness for downstream counting and sizing); direct comparisons on such benchmarks cannot effectively validate the depth-specific design choices of this pipeline. Cross-device validation on additional industrial depth sensors remains the more informative next step. Second, the dataset remains private, although we partially mitigate this issue by planning to release, upon acceptance, the evaluation code, the fixed file lists, the released test-set contour masks, the test-set probability maps, and the trained checkpoints. Third, the isolated gain of the gated fusion block is modest, so we do not claim that this single module is the dominant innovation. Fourth, the current tolerance study is specific to the present line-scan depth-imaging setup and one-pixel annotation protocol rather than a universal statement for all sensors or annotation protocols. Fifth, although the proposed method remains competitive on External130, DexiNed remains slightly ahead in ODS/AP on that split, indicating that cross-session robustness and score calibration can still be improved. Sixth, the External130 evaluation does not explicitly cover several dynamic environmental disturbances commonly encountered in industrial conveyor systems, such as airborne dust, moisture on particle surfaces, and mechanical vibration. This is an important limitation because the proposed input representation includes high-frequency geometric priors, especially Sobel gradient magnitude and Laplacian response, which may be sensitive to surface noise, missing depth values, local scanning artifacts, and vibration-induced profile fluctuations. Seventh, the encoder is initialized from ImageNet-pretrained MiT-B0 weights, whereas the input channels are deterministic geometric measurements rather than natural RGB textures. This input-domain gap may limit the ability of the pretrained encoder to extract depth-specific boundary features. Future work will therefore explore domain-specific or self-supervised pretraining directly on conveyor-belt depth maps, as well as adaptive denoising, confidence-weighted geometric priors, and uncertainty-aware fusion for improved robustness under environmental disturbances. Future work will evaluate the proposed design on additional sensors and materials, perform separately controlled tests for belt speed, measuring rate, dust, moisture, and vibration, explore domain-specific or self-supervised pretraining directly on depth modalities, explore broader public benchmarking where possible, and integrate contour extraction more tightly with downstream instance segmentation and geometric measurement pipelines.

## Figures and Tables

**Table 1:** Acquisition conditions for the main split and the external test-only split.

Set	Sensor Model	Session	Exposure	Measuring Rate	Belt Speed	Usage
Main split	Wenglor MLSL276	A	150 µs	200 Hz	≈0.21 m/s	train/val/test
External130	Wenglor MLSL276	B	150 µs	250 Hz	≈0.26 m/s	test-only

**Table 2:** Quantitative comparison on AGG_FULLDATA under tolerance matching (tol=1 px).

Method	ODS	OIS	AP	BestThr
Ours (HybridMiT)	**0.9607**	**0.9716**	**0.9683**	0.74
DexiNed	0.9578	0.9658	0.9677	0.30
BDCN (VGG16)	0.9485	0.9548	0.9371	0.95
HED (VGG16)	0.9473	0.9515	0.9558	0.29
Baseline (MiT)	0.9406	0.9512	0.9411	0.69
RCF (VGG16)	0.9354	0.9389	0.9232	0.94
U-Net (ResNet-50)	0.9276	0.9352	0.9217	0.52
TEED	0.9191	0.9240	0.9433	0.87
PiDiNet	0.9152	0.9196	0.9159	0.95
Canny	0.7675	0.7646	0.5926	0.01

Note: Bold values indicate the best value in each metric column.

**Table 3:** Strict pixel-exact reference on AGG_FULLDATA (tol=0 px).

Method	ODS	OIS	AP	BestThr
Ours (HybridMiT)	**0.6476**	**0.6679**	0.5735	0.64
DexiNed	0.6399	0.6502	**0.5977**	0.29
HED (VGG16)	0.6148	0.6116	0.5739	0.28
BDCN (VGG16)	0.6130	0.6118	0.5117	0.93
Baseline (MiT)	0.6039	0.6134	0.5104	0.62
RCF (VGG16)	0.5905	0.5843	0.4916	0.93
U-Net (ResNet-50)	0.5825	0.5838	0.4630	0.31
TEED	0.5720	0.5747	0.5070	0.87
PiDiNet	0.5623	0.5562	0.4634	0.94
Canny	0.4019	0.4042	0.1669	0.01

Note: Bold values indicate the best value in each metric column.

**Table 4:** DexiNed input-sensitivity analysis. Rep3 denotes replicated single-channel depth input; DSL3 denotes the same Depth+Sobel+|Laplacian| pseudo-RGB input used by HybridMiT.

Variant	tol=1			tol=0		
	ODS	OIS	AP	ODS	OIS	AP
DexiNed (Rep3)	**0.9578**	**0.9658**	**0.9677**	**0.6399**	**0.6502**	**0.5977**
DexiNed (DSL3)	0.9431	0.9473	0.9541	0.6097	0.6072	0.5603

Note: Bold values indicate the better value between the two DexiNed input variants for each metric column.

**Table 5:** Summary of the rigid-object repeatability study. Mean and P95 distances are measured in pixels between each aligned scan boundary and the category consensus boundary using bidirectional nearest-boundary distances. The size-stability column reports the coefficient of variation (CV) of the equivalent diameter across repeated scans.

Object	Mean dist. (px)	P95 dist. (px)	Within-0 px (%)	Within-1 px (%)	Within-2 px (%)	Eq. Diameter CV (%)
Triangle	0.452	1.414	61.7	94.8	98.3	0.88
Cylinder	0.301	1.000	72.6	97.2	100.0	1.09
Cube	0.219	1.000	78.2	99.8	100.0	0.81
Overall	0.335	1.000	70.1	97.1	99.4	0.93

**Table 6:** Cross-session evaluation on the External130 same-sensor test-only split acquired under altered operating conditions. Methods are ordered by ODS under the primary protocol (tol=1).

Method	tol=1			tol=0		
	ODS	OIS	AP	ODS	OIS	AP
DexiNed	**0.9607**	0.9694	**0.9714**	**0.6494**	0.6526	**0.6139**
Ours (HybridMiT)	0.9580	**0.9734**	0.9683	0.6480	**0.6697**	0.5747
HED (VGG16)	0.9472	0.9541	0.9560	0.6179	0.6100	0.5779
Baseline (MiT)	0.9389	0.9537	0.9429	0.6047	0.6112	0.5106
TEED	0.9170	0.9281	0.9433	0.5704	0.5742	0.5064
PiDiNet	0.9119	0.9215	0.9152	0.5602	0.5515	0.4610

Note: Bold values indicate the best value in each metric column.

**Table 7:** Efficiency comparison measured on a single NVIDIA GeForce RTX 4060 (8 GB) with input resolution 832×576 under single-image FP32 inference. Latency and FPS are averaged after warm-up using GPU event-based timing with device synchronization. Peak allocated GPU memory is reported as the maximum memory allocated by the runtime during inference (excluding cached/reserved memory and data-loading overhead). FLOPs are estimated as 2× MACs using a standard FLOP-counting tool and may not account for all implementation-specific operators (e.g., interpolation or fused kernels).

Method	Params (M)	Est. FLOPs (G)	FPS	Latency (ms)	Peak alloc. VRAM (GB)
Ours (HybridMiT)	3.71	37.09	48.9	20.45	0.27
Baseline (MiT)	3.53	20.86	74.7	13.39	**0.17**
TEED	**0.06**	**7.66**	**199.0**	**5.02**	0.20
PiDiNet	0.69	71.37	19.1	52.46	0.67
HED (VGG16)	14.72	293.27	30.9	32.32	0.39
RCF (VGG16)	14.72	293.27	30.9	32.35	0.37
BDCN (VGG16)	16.30	526.19	13.4	74.58	1.08
DexiNed	35.08	510.51	20.1	49.81	0.48
U-Net (R50)	73.68	732.09	13.4	74.72	1.07

Note: Bold numeric values indicate the best value in each efficiency column; for FPS, higher is better, whereas for parameters, FLOPs, latency, and peak allocated VRAM, lower is better.

**Table 8:** Key training settings for AGG_FULLDATA.

Item	AGG_FULLDATA
Batch size	4
Optimizer	AdamW
Learning rate	$2\times 10^{-4}$
Weight decay	0.05
Max training steps	30,000
Min training steps	6000
Early-stopping patience	6000
Validation interval	every 1000 steps
Context encoder	MiT-B0 (SegFormer), pre-trained
Data augmentation	random flips
Auxiliary supervision	enabled (λ=0.4)
Threshold sweep	0.01–1.01, step 0.01
Model selection	validation ODS under tol=1; report tol∈{0,1}
Random seed	42

**Table 9:** Ablation study on AGG_FULLDATA under the same protocol. We report both tolerance matching (tol=1) and strict pixel-exact reference (tol=0).

Variant	tol=1			tol=0		
	ODS	OIS	AP	ODS	OIS	AP
Baseline (MiT-only)	0.9406	0.9512	0.9411	0.6039	0.6134	0.5104
Without geometric priors	0.9509	0.9614	0.9581	0.6263	0.6390	0.5489
Without local branch	0.9525	0.9629	0.9610	0.6307	0.6439	0.5557
Without gate	0.9592	0.9707	0.9673	0.6448	0.6654	**0.5736**
Without full-resolution refinement	0.9449	0.9553	0.9465	0.6104	0.6206	0.5187
Proposed (full)	**0.9607**	**0.9716**	**0.9683**	**0.6476**	**0.6679**	0.5735

Note: Bold values indicate the best value in each metric column.

## Data Availability

Due to project and data-sharing agreements, the raw AGG_FULLDATA and External130 depth maps cannot be publicly released. Upon acceptance, we will release a reproducibility archive containing: (i) the full evaluation codebase; (ii) the fixed file lists for the AGG_FULLDATA and External130 test sets; (iii) the released test-set contour masks for these two evaluation splits; (iv) the pre-computed probability maps for our method and the compared baselines on these test sets; and (v) the trained checkpoints for HybridMiT and the compared learning-based baselines. This archive is intended to allow the community to verify our metrics and benchmark against our reported results without requiring access to the proprietary raw data. Access to the remaining full dataset, including unreleased depth maps and annotations outside the released test splits, can be provided to qualified researchers under a non-disclosure agreement.
